# Reinitiating Chemotherapy beyond Progression after Maintenance Immunotherapy in Extensive-Stage Small-Cell Lung Cancer

**DOI:** 10.3390/medicina60081225

**Published:** 2024-07-28

**Authors:** Roxana-Andreea Rahnea-Nita, Radu-Valeriu Toma, Valentin Titus Grigorean, Ionuţ Simion Coman, Violeta Elena Coman, Iancu Emil Pleşea, Anwar Erchid, Gabriel-Petre Gorecki, Gabriela Rahnea-Nita

**Affiliations:** 1 8th Clinical Department—Radiology, Oncology and Hematology, Faculty of Medicine, “Carol Davila” University of Medicine and Phamacy, 020021 Bucharest, Romania; roxana.rahnea-nita@umfcd.ro (R.-A.R.-N.); radu.toma@umfcd.ro (R.-V.T.); 2 Department of Oncology-Palliative Care, “Sf. Luca” Chronic Diseases Hospital, 041915 Bucharest, Romania; gabriela.rahnea-nita@umfcd.ro; 3 Department of Radiotherapy, “Prof. Dr. Alexandru Trestioreanu” Oncological Institute, 022328 Bucharest, Romania; 4 10th Clinical Department—General Surgery, Faculty of Medicine, “Carol Davila” University of Medicine and Pharmacy, 020021 Bucharest, Romania; ionut.coman@umfcd.ro (I.S.C.); elena.coman@umfcd.ro (V.E.C.); 5 Department of General Surgery, “Bagdasar-Arseni” Clinical Emergency Hospital, 041915 Bucharest, Romania; erchid.anwar@yahoo.com; 6 Department of Histopathology, “Bagdasar-Arseni Clinical Emergency Hospital”, 041915 Bucharest, Romania; pie1956@yahoo.com; 7 Department of Anesthesia and Intensive Care, Faculty of Medicine, “Titu Maiorescu” University, 031593 Bucharest, Romania; gabriel.gorecki@prof.utm.ro; 8 Department of Anesthesia and Intensive Care, CF2 Clinical Hospital, 011464 Bucharest, Romania; 9 Specific Disciplines Department, Faculty of Nursing and Midwifery, “Carol Davila” University of Medicine and Pharmacy, 020021 Bucharest, Romania

**Keywords:** small-cell lung cancer, first-line immunotherapy, reinitiating chemotherapy, Epoetinum alpha

## Abstract

*Introduction*: Small-cell lung cancer (SCLC) is an aggressive form of cancer with a poor prognosis. The two-year survival rate is 8% of all cases. *Case presentation*: We present the case of a male patient who was 50 years old at the time of diagnosis in May 2022. He was diagnosed with extensive-stage small-cell lung cancer, treated with immunotherapy in combination with chemotherapy (Durvalumab in combination with Etoposide plus Carboplatin) as a first-line treatment, followed by maintenance immunotherapy. In December 2023, a PET-CT scan revealed progressive disease with multiple metastases. Chemotherapy was reinitiated with Etoposide plus Cisplatin in January 2024. After two cycles of chemotherapy, the patient developed post-chemotherapy anemia, for which treatment with Epoetinum alpha was initiated. Chemotherapy was continued for another five cycles, until May 2024, with the maintenance of hemoglobin at a level within 9.9 mg/dL–11 mg/dL. Upon assessment at the end of May 2024, the patient presented an ECOG = 2 performance status, with a moderate general state, moderate-intensity fatigue, no pain, no anxiety or depression and no dyspnea. *Discussions, Literature Review and Conclusions*: Reinitiating chemotherapy after the failure of maintenance immunotherapy may be an option in patients with SCLC. Epoetinum allows oncological treatment by preventing chemotherapy-induced anemia.

## 1. Introduction

Small-cell lung cancer (SCLC) is an aggressive form of cancer with a poor prognosis. SCLC represents 15% of all lung cancers, and it occurs in advanced stages (extensive stage of the disease) in 70% of cases. Metastasis has already occurred in most patients at the time of diagnosis. The overall two-year survival rate is 8% of all cases. The origin of this type of cancer is neuroendocrine, and long-term smoking is a proven risk factor in this type of cancer [1,2,3,4,5,6,7].

The first standard line of chemotherapy consists of platinum salts plus Etoposide, and the second line is Topotecan treatment.

In recent years, however, immune checkpoint inhibitors (ICI) have become the first-line therapy in extensive-stage small-cell lung cancer (ES-SCLC) [8,9]. Thus, first-line Durvalumab in combination with Etoposide plus Cisplatin or Carboplatin (EP) has significantly improved the overall survival (OS) compared to Etoposide plus Cisplatin or Carboplatin alone in ES-SCLC, with a mean survival rate of 12.9 months vs. 10.5 months [10].

Durvalumab plus EP treatment is administered in four series, every 21 days, and it is followed by maintenance immunotherapy with Durvalumab at 28-day intervals. 

In non-small-cell lung cancer (NSCLC), there are few previous studies on second-line chemotherapy after immunotherapy [11].

Epoetinum alpha has the role of increasing the level of hemoglobin in cancer patients who develop chemotherapy-associated anemia [12]. The administered dose is 40,000 units, once a week.

The peculiarity of the case presentation in this article is the reinitiation of chemotherapy after the progression of the disease through multiple metastases 18 months after the diagnosis, with the association of erythrocyte and leukocyte growth factors, which allowed the maintenance of the chemotherapeutic doses and the time interval between the administration of the chemotherapeutic series, with the OS being 24 months. 

## 2. Case Presentation

We present the case of a male patient who was 50 years old at the time of diagnosis in 2022. 

The patient worked as a boiler attendant for 11 years; then, he worked as a security guard. He has currently been retired for 2 years due to the illness.

The patient was a smoker from the age of 10 to the age of 50—he smoked 40 cigarettes/day. He quit smoking in 2022 after he became sick. 

In May 2022, the patient went to the Oncology–Palliative Care Department of “St. Luca” Chronic Disease Hospital in Bucharest, after a fibrobronchoscopy with endobronchial biopsy of the right lung, performed in May 2022 at the Emergency Teaching Hospital in Bucharest. The histopathological and immuno-histochemical examinations led to the diagnosis of a small-cell neuroendocrine carcinoma. After the evaluation, the diagnosis was made: a right bronchopulmonary neoplasm—multiple tumors (SCLC) stage 3C. 

In terms of comorbidities, the patient exhibited recently diagnosed diabetes and second-degree obesity. The patient underwent treatment with oral antidiabetic medication for his diabetes. 

The first-line treatment was initiated in May 2022, with immunotherapy (Durvalumab 1500 mg every 21 days) plus Etoposide plus Carboplatin (EP) every 21 days, for four series, followed by maintenance therapy with Durvalumab since July 2022, 1500 mg every 28 days. 

**Figure 1:**
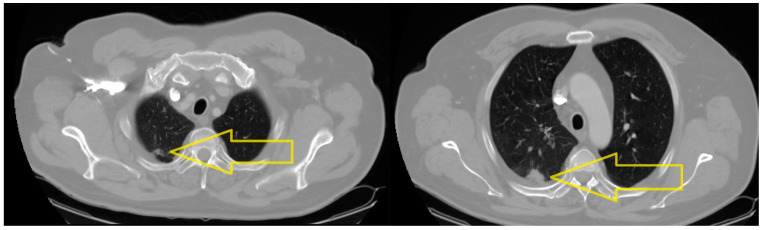
CT scan chest (October 2022): An expanding nodular mass in the apical segment of the right upper lobe with maximum axial diameters of 1.7/0.7. An expanding nodular mass plated in the posterior pleura of the posterior segment of the right upper lobe with maximum axial diameters of 2.2/1.5 mm.

The CT scan of the head, chest, abdomen and pelvis, performed in October 2022, revealed the following: an expanding nodular mass in the apical segment of the right upper lobe with maximum axial diameters of 1.7/0.7 mm (in regression from 2.6/1.9 mm); an expanding nodular mass plated in the posterior pleura of the posterior segment of the right upper lobe with maximum axial diameters of 2.2/1.5 mm (in regression from 4.9/3.6 mm). Conclusion: The CT scan revealed a partial improvement from the previous examination (since May 2022, when the diagnosis was made), with the dimensional regression of the right pulmonary nodular lesions and of the mediastinal adenopathies (Figure 1).

Maintenance therapy with Durvalumab was continued until March 2023, when a head CT scan and a cranial MRI were performed. 

**Figure 2:**
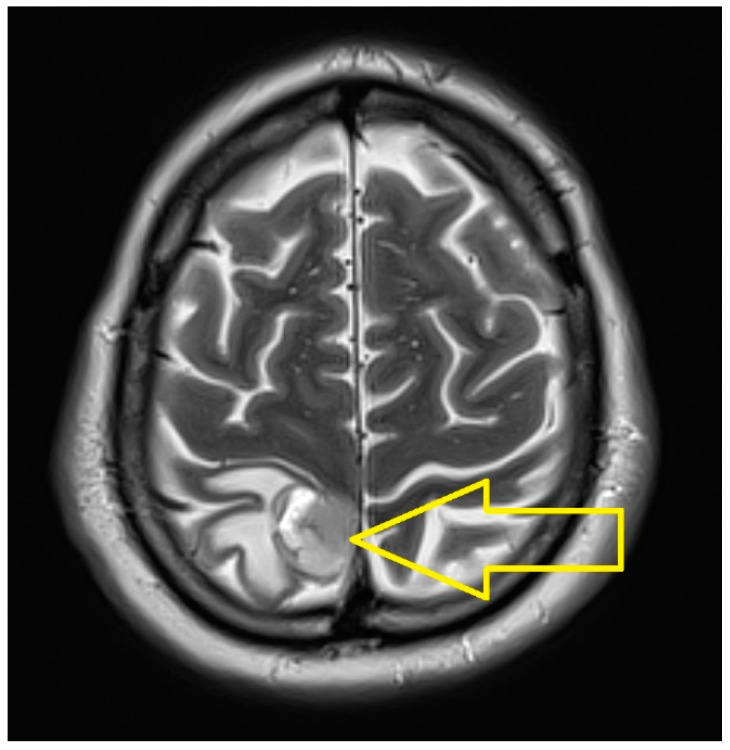
The cranial MRI (March 2023): Right parietal nodule that is compatible on imaging with a secondary hematogenous mass with intralesional bleeding.

The cranial MRI revealed a right parietal nodule that was compatible on imaging with a secondary hematogenous mass with intralesional bleeding, for which a Gamma Knife examination was recommended, which subsequently recommended a whole brain radiotherapy examination (Figure 2).

In April 2023, external radiation was performed using the IMRT-VMAT technique (arch-therapy with intensity volume modulation), at the level of the following target volumes: cerebral—“whole brain” DT = 30 Gy in 10 fractions for 2 weeks. 

After this oligo-progression (through brain metastasis) in March 2023, Durvalumab immunotherapy was continued from May 2023 until December 2023.

In June 2023, the patient underwent a cerebral MRI and CT scan of the chest and abdomen.

**Figure 3:**
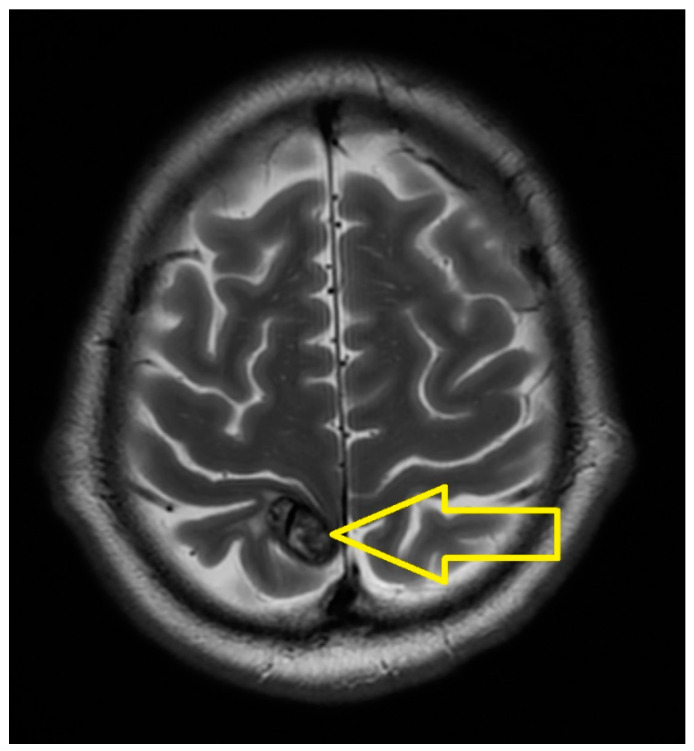
Cerebral MRI (June 2023): Dimensional regression of the right parietal tumor mass and of the perilesional edema.

The cerebral MRI revealed the dimensional regression of the right parietal tumor mass and of the perilesional edema (Figure 3). 

**Figure 4:**
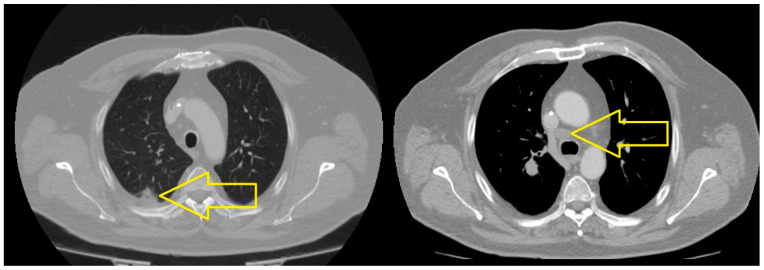
Chest CT scan (June 2023): A small nodular tissue lesion in the pulmonary parenchyma in the posterior segment of the right upper lobe with axial diameters of 18/15 mm and a dense tissular micro-nodular lesion located above the right posterior hilum with axial diameters of 17/15 mm. A small nodular lesion located in the right posterior apex with axial diameters of 9.6 mm The pulmonary lesions exhibit minimal regression from the previous CT scan. Several mediastinal adenopathies with diameters of up to 22 mm and in the right pulmonary hilum with diameters of up to 30/26 mm.

The thoracic CT scan revealed a small nodular tissue lesion in the pulmonary parenchyma in the posterior segment of the right upper lobe with axial diameters of 18/15 mm and a dense tissular micro-nodular lesion located above the right posterior hilum with axial diameters of 17/15 mm. A small nodular lesion was located in the right posterior apex, with axial diameters of 9.6 mm. The pulmonary lesions exhibited minimal regression from the previous CT scan. Several mediastinal adenopathies were seen with diameters of up to 22 mm and in the right pulmonary hilum with diameters up to 30/26 mm (Figure 4).

The abdominal CT scan revealed the increased dimensions of the liver, without space replacing the masses. No secondary bone determinations were visible on the CT scan in the examined areas. 

It should be mentioned that in November 2023, laboratory tests revealed liver cytolysis, which was remitted under hepatoprotective treatment.

**Figure 5:**
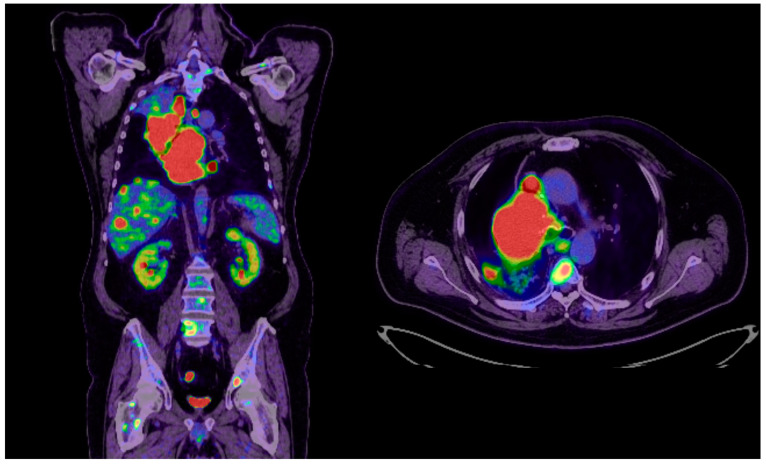
PET-CT (December 2023): A bulky tumor mass in the right mediastinal–pulmonary area that includes the right main bronchus, with maximum dimensions of 13.4/10.2/8.5. Multiple bilateral pulmonary micronodules. Liver with greatly increased dimensions, almost completely occupied by multiple nodular and macronodular lesions, the largest with dimensions of 68/45 mm, compatible with secondary determinations. Two lesions in the pancreas, the largest of 25/20 mm. Bulky adenopathies in the celio-mesenteric and retrocrural areas, the largest inferior to the perigastric cardia and 25/20 mm in dimension. The skeletal system: multiple metabolically active lesions. Disseminated metastatic oncologic disease with secondary determination in the lungs, supra- and subdiaphragmatic ganglia, liver, pancreas, muscles and bones.

The PET-CT scan performed in December 2023 highlighted the following: left supraclavicular adenopathy; a bulky tumor mass in the right mediastinal–pulmonary area that included the right main bronchus, with maximum dimensions of 13.4/10.2/8.5, which was metabolically active and determined complete right upper lobe atelectasis; a carcinomatous lymphangitis appearance; multiple bilateral pulmonary micronodules; minimal right pleurisy; a liver with greatly increased dimensions, almost completely occupied by multiple nodular and macronodular lesions, the largest with dimensions of 68/45 mm, compatible with secondary determinations; two lesions in the pancreas, the largest of which had dimensions of 25/20 mm; bulky adenopathies in the celio-mesenteric and retrocrural areas, the largest inferior to the perigastric cardia and 25/20 mm in dimension; an intramuscular paravertebral tissular mass, on the right side, at the level of L2, with maximum dimensions of 25/23 mm. The skeletal system exhibited multiple metabolically active lesions, disseminated at the level of the cervical–thoracic–lumbar–sacral spine, the rib cage, the sternum, the bilateral clavicles, the right humeral head, the bones of the pelvis and the bilateral proximal femur. The PET-CT scan indicated disseminated metastatic oncologic disease with secondary determination in the lungs, supra- and subdiaphragmatic ganglia, liver, pancreas, muscles and bones (Figure 5). 

In December 2023, the performance status of the patient was ECOG = 3.

In January 2024, it was decided to stop immunotherapy with Durvalumab and restart chemotherapy with Etoposide and Cisplatin (instead of Carboplatin, which had been administered at the beginning of the treatment, between May and July 2022). A total of seven cycles of chemotherapy with Etoposide plus Cisplatin were administered between January and May 2024.

It was our therapeutic decision to reinitiate the EP treatment plan, replacing Carboplatin with Cisplatin, given the favorable response to this therapeutic plan revealed by the CT scan in October 2022.

During the first two series of chemotherapy, in January 2024, the hemoglobin level was within normal limits, but, in February 2024, during the third chemotherapy series, the hemoglobin level was 9.9 mg/dL. For this reason, starting with the fourth series, in March 2024, treatment with Epoetinum alpha was initiated, at 40,000 units per week, for the treatment of chemotherapy-induced anemia. In the following three chemotherapy series (cycles 5–7), the patient continued the treatment with Epoetinum alpha, the hemoglobin level being 10.9 mg/dL in March 2024 (cycle 4), 9.9 mg/dL in April 2024 (cycle 5) and 11 mg/dL and 10.7 mg/dL in May 2024 (cycles 6 and 7).

After each chemotherapy cycle, long-acting granulocyte colony-stimulating factor (Pegfilgrastim) was administered subcutaneously in a 6 mg dose, 24 h from chemotherapy administration.

After seven series of chemotherapy were administered, the patient was assessed in terms of physical and psycho-emotional symptoms at the end of May 2024, using the Edmonton Symptom Assessment System (ESAS) [13,14,15] and the Hospital Anxiety and Depression Scale (HADS) [16,17,18].

The ESAS is a visual analogue scale, being a valid and reliable tool that assesses nine common symptoms experienced by cancer patients: pain, tiredness, drowsiness, nausea, lack of appetite, depression, anxiety, shortness of breath and impaired well-being. The patient is instructed to assess the severity of each symptom on a scale from 0 to 10, where 0 represents the absence of symptoms and 10 represents the worst possible severity. The ESAS scores 0, 1–3, 4–6 and 7–10 points on a scale from 0 to 10 generally correspond to none, mild, moderate and severe symptom burdens. The ESAS is freely available.

The HADS is a 14-item self-report measurement tool that assesses anxiety and depression. It uses a four-point scale, ranging from 0 (which means not at all) to 3 (which means very often indeed). The responses provide separate scores for anxiety and depression (the HADS produces two subscales, one for anxiety (HADS–A) and one for depression (HADS–D)). The total score is 21 per subscale. Anxiety or depression scale has a score ranging from 0 to 21. The total score is as follows: 0–7 = normal, 8–10 = borderline and 11–21 = abnormal. The HADS is free and available online.

The results were as follows.

Regarding the ESAS:

- 0 points—pain, shortness of breath, anxiety, depression, other symptoms;
- 1–3 points—no symptoms;
- 4–6 points—fatigue, drowsiness, nausea, loss of appetite, worst possible feeling of well-being, other symptoms (visual impairments);
- 7–10 points—no symptoms.

Regarding the HADS:

- Anxiety—2 points—normal;
- Depression—7 points—normal.

The evaluation of the physical symptoms revealed fatigue, drowsiness, nausea, a loss of appetite, the worst possible well-being and other symptoms (visual impairment) of moderate intensity, which were treated symptomatically (antiemetic drugs, corticosteroids), while the assessment of the psychological symptoms did not reveal anxiety or depression.

Moreover, regarding the performance status, the patient had a performance status of ECOG = 2 at the end of May 2024. 

In June 2024, we recommended a CT scan for evaluation.

**Figure 6:**
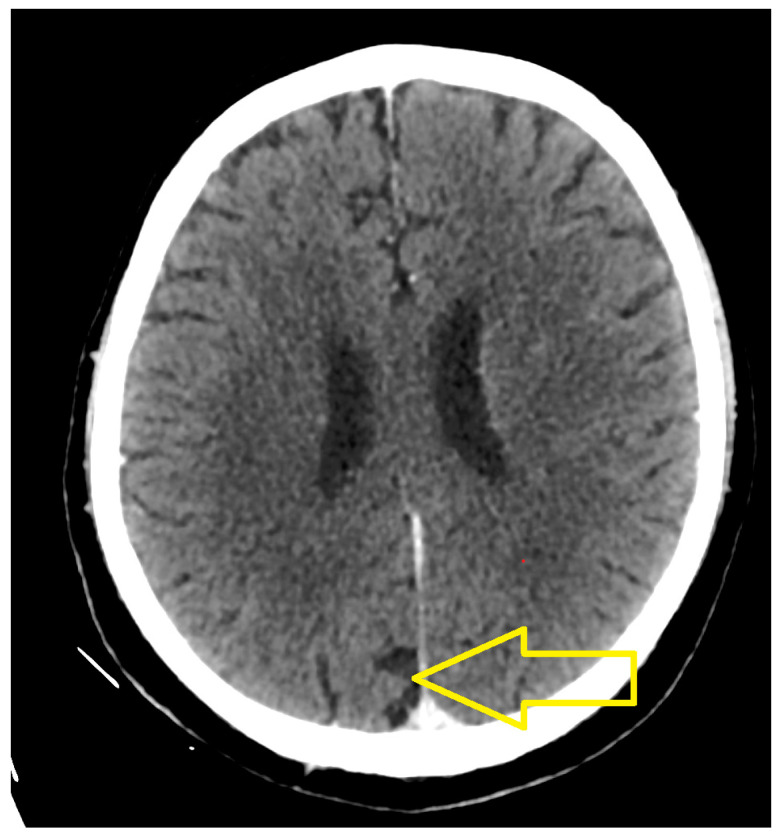
Skull CT (June 2024): At the cerebral level, in the right para-sagittal parietal vertex, a secondary brain metastasis is evident, in regression compared to March 2023.

**Figure 7:**
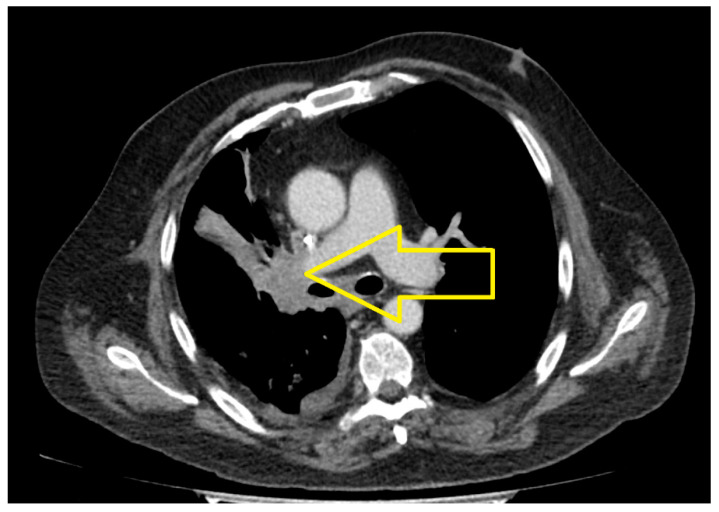
Chest CT (June 2024): Lung metastases located apically and right supra-hilar, with maximum axial diameters of 85/22 mm and 42/20 mm, respectively, in progression compared to June 2023.

**Figure 8:**
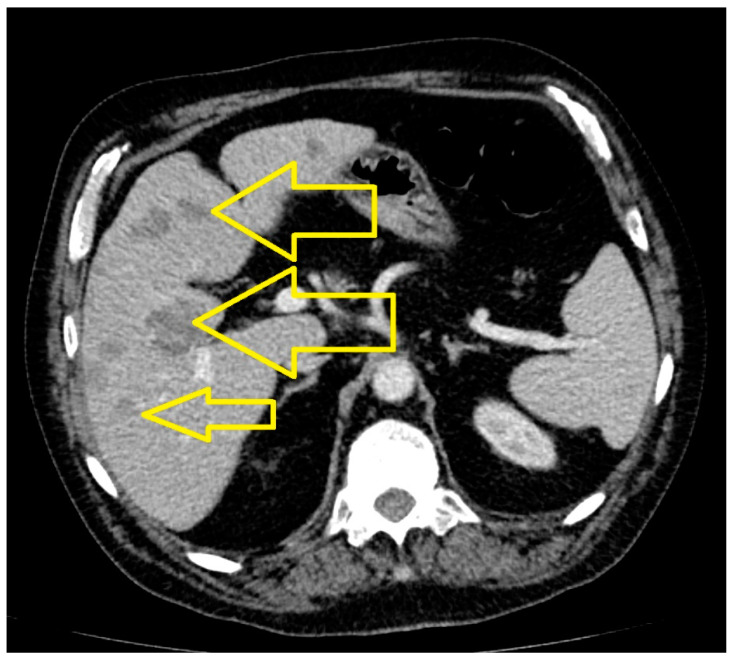
Abdominal CT (June 2024): Liver with slightly increased dimensions, with multiple liver metastases and with a diameter of up to 35 mm.

The results highlighted the following. At the cerebral level, in the right para-sagittal parietal vertex, a secondary determination was evident, in regression compared to March 2023. At the level of the pulmonary parenchyma, condensing lesions were evident, located apically and right supra-hilar, with maximum axial diameters of 85/22 mm and 42/20 mm, respectively, in progression compared to June 2023. The liver had slightly increased dimensions, with a steatotic structure, multiple hypodense, hypocapturing lesions and the appearance of secondary determinations with a diameter of up to 35 mm (Figure 6, Figure 7 and Figure 8).

According to the recommendations of the Multidisciplinary Oncological Commission, which met in June 2024, second-line monochemotherapy with Paclitaxel was initiated.

In June 2024 (the date that this article was written), the patient had a performance status of ECOG = 2 and a level of hemoglobin = 9.1 mg/dL and had received the first cycle of second-line monochemotherapy at the time of writing.

## 3. Discussion and Literature Review

We reviewed the most relevant articles recently published and we selected ten studies with a clinical impact, published in the years 2023 and 2024.

The main updates included the predictors of survival, symptoms and experiences, chemotherapy, radiotherapy and immunotherapy.

The selected articles are synthesized and listed in Table 1.

SCLC ranges from the limited stage (LS-SCLC) to the extensive stage (ES-SCLC), with a pragmatic basic approach, thus classifying patients according to the therapeutic strategy and whether it includes radiotherapy or not [19].

Around 60–70% (two out of three) of the patients with SCLC are diagnosed in the advanced stages, and approximately 95% of the patients in the extensive stages have already experienced metastasis at the moment of diagnosis [4,5,20,21,22].

In a retrospective study conducted in Canada, between 2004 and 2018, on a total of 537 patients with ES-SCLC, David E. Dawe et al. assessed the overall survival rate at 1 year, 2 years and 5 years depending on the performance status. In patients with ECOG = 0, the OS was 17% in 2 years; in patients with ECOG = 1–3, the OS was 8% in 2 years; and in patients with ECOG = 3–4, the OS was 3% in 2 years. The unfavorable prognostic factors were brain and hepatic metastases, high lactate dehydrogenase (LDH), low hemoglobin levels and abnormal sodium levels. Only 56.1% of the patients included in the study received Cisplatin and 45.6% underwent radiotherapy of the thorax. Very few patients underwent prophylactic cranial radiation [1].

In a study conducted in 2021 on nine patients with ES-SCLC, D. Gwyn Bebb et al. highlighted that the symptoms with the strongest impact on the patients were shortness of breath, fatigue, coughing, chest pain and nausea/vomiting [23]. The conclusion of this study is that healthcare professionals should understand both the priorities of the patients and their opinions in order to ensure that the patients will benefit from the most appropriate treatment [23] that will grant them the best quality of life possible [24].

However, paraneoplastic syndromes are most frequently associated with small-cell lung cancer (SCLC) [25].

Minimal progress has been achieved in the treatment of SCLC in the past few decades, with the standard first-line treatment for ES-SCLC in the past 30 years being chemotherapy (platinum doublet chemotherapy with either Cisplatin or Carboplatin in combination with Etoposide as the first-line regimen and Topotecan as the second-line regimen) [26,27].

Immune checkpoint inhibitors (ICI), namely programmed death receptor-1 (PD-1) antibodies (Nivolumab, Pembrolizumab), the monoclonal antibody against PD-L1 (Atezolizumab) and cytotoxic T-lymphocyte-associated antigen 4 (CTLA-4) (Ipilimumab, Tremelimumab) have proven their activity potential against cancer cells by stimulating the immune system, being able to prolong the OS in patients with SCLC [7,28,29].

Immunotherapy was approved both as a first-line therapy in metastatic SCLC and as a third-line therapy in metastatic SCLC after the failure of two chemotherapy regimens, being approved by the Food and Drug Administration (FDA). Specifically, this included four drugs: Pembrolizumab, Nivolumab, Atezolizumab and Durvalumab [27].

There are ongoing studies to predict the efficacy of ICI. Da Hyun Kang et al., in a prospective multicenter study in patients with advanced non-small-cell lung cancer, calculated the immune checkpoint inhibitor score (IChIS) by analyzing the blood cell characteristics (sum of neutrophil count score and immature granulocyte score) and concluded that this score could be a biomarker for the prediction of a survival benefit in NSCLC patients [30].

While, in the immunotherapy era, the first-line chemo-immunotherapy is the standard in the care of ES-SCLC, the second-line treatment options are limited. Among them, anti-PD-1 and anti-PD-L1 monoclonal antibodies and Delta-like ligand 3 (DLL3) have been evaluated alone or in combination [31].

Many patients may experience disease recurrence after immunotherapy, and, in this case, second- and third-line chemotherapy may play an important role, leading to better response rates and increased OS [10,32,33].

Epoetinum alpha has the role of improving the quality of life in patients undergoing chemotherapy [34], increasing the level of hemoglobin, reducing fatigue and increasing the ability to perform typical daily activities [35]. At the same time, erythropoietins prevent severe anemia and reduce the administration of transfusions in patients with a high risk of developing post-chemotherapy anemia [35].

**Table 1:** Comparative analytical data.

No.	Author—Year	Subject	Reference No.
1	Dawe DE—2023	Predictors of survival	[1]
2	Wang Q—2023	Epidemiology	[4]
3	Basumallik N—2024	Current treatments	[5]
4	Saida Y—2023	Current landscape and future	[8]
5	Assi HI—2023	Chemotherapy post-immunotherapy	[11]
6	Bebb DG—2023	Symptoms and experiences	[23]
7	Rahnea-Nita RA—2023	Efficacy of immunotherapy	[25]
8	Rahnea-Nita RA—2024	Immunotherapy continued beyond progression	[29]
9	Meriggi F—2024	Second-line treatment options	[31]
10	Hirata T—2023	Second-line chemotherapy after immunotherapy	[33]

There is a correlation between the hemoglobin level and quality of life. Erythropoietins are effective and safe in the treatment of anemia, increasing and maintaining the hemoglobin levels and reducing the transfusion requirements [35].

The presence of anemia has a negative impact on the quality of life of patients with cancer [36]. Two studies have demonstrated improvements in quality of life through the treatment of anemia with erythropoiesis-stimulating proteins in patients with cancer [36]. 

Littlewood T.J et al., in a randomized placebo-controlled trial that included 375 patients with non-myeloid hematologic malignancies, who were treated with epoetin alfa, reported a significant increase in hemoglobin levels and a significant improvement in energy levels, activity and quality of life [37]. Hudis et al., in an open-label, non-randomized, multicenter study in patients with stage I–III breast cancer, with baseline hemoglobin ≥ 10 to ≤12 g/dL, reported also significant improvements in energy, activity and quality of life after 12 weeks of epoetin alfa therapy [38].

Thomas Grote et al., in a randomized, double-blind, placebo-controlled trial on patients with small-cell lung cancer, evaluated the effects of epoetin alfa on survival and on the response to chemotherapy. The conclusion of the study was that the overall tumor response and overall survival were similar between the epoetin alfa and placebo groups after three chemotherapy cycles. Epoetinums are not recommended in cancer patients with hemoglobin levels >12 g/dL, because of the risk of thrombovascular events [36,39].

This study focused on a male patient who had smoked for 40 years, since the age of 10, who developed SCLC at the age of 50, with an initially favorable response, with progression-free survival (PFS) of 10 months, after first-line Durvalumab in combination with Etoposide plus Carboplatin, followed by maintenance immunotherapy, with the occurrence of oligoprogression after 10 months due to already radiated brain metastasis. This was continued with maintenance immunotherapy and followed by the reinitiation of chemotherapy after progression due to disseminated metastatic oncologic disease.

Moreover, after each chemotherapy cycle, Pegfilgrastim was administered, for the primary prophylaxis of febrile neutropenia [40].

Moreover, we mention that, upon the initiation of treatment in May 2022, the performance status of the patient was ECOG = 2, which became ECOG = 3 in December 2023, along with the progression of the disease, with the occurrence of multiple metastases; it then became ECOG = 2 again in May 2024, after reinitiating chemotherapy.

## 4. Conclusions

Although chemotherapy with Etoposid and platinum salts was considered the only standard care treatment for SCLC for decades, and although SCLC produces a sensitive response to chemotherapy, the overall survival rate is only 10 months in most patients.

Over the past decade, immunotherapy has led to significant progress in the treatment of these patients, with chemo-immunotherapy being the preferable initial treatment for advanced SCLC.

However, the survival rate is still low in SCLC patients.

Reinitiating chemotherapy after failure to maintain immunotherapy may be an option in patients with SCLC.

Erythropoietin allows oncological treatments to prevent anemia and improve the quality of life in cancer patients.

Further research into personalized strategies for SCLC patients is, however, required.

## Data Availability

Data is available by request to the first author.
